# A meta-analysis of narrow-band imaging for the diagnosis of primary nasopharyngeal carcinoma

**DOI:** 10.12688/f1000research.15183.1

**Published:** 2018-06-18

**Authors:** David CM Yeung, Alexander C Vlantis, Eddy WY Wong, Michael CF Tong, Jason YK Chan

**Affiliations:** 1 Department of Otorhinolaryngology, Head & Neck Surgery, Prince of Wales Hospital, Chinese University of Hong Kong, Shatin, New Territories , Hong Kong

**Keywords:** Nasopharyngeal carcinoma, narrow-band imaging, endoscopy, meta-analysis

## Abstract

**Background**: Narrow band imaging (NBI), an endoscopic technique featuring an augmented definition of microvasculature and mucosal patterns. NBI is increasingly advocated as a tool to characterize neoplasia and intestinal metaplasia in endoscopic standards, such as for colorectal polyps and tumors. Recently NBI has also been studied in the detection of Nasopharyngeal Carcinoma (NPC). Here we aimed to assess the diagnostic utility of NBI for the diagnosis of NPC.

**Methods: **A meta-analysis of studies comparing narrow-band imaging and white light endoscopy in the diagnosis of primary nasopharyngeal carcinoma was performed. The review process involved two independent investigators. The databases used were MEDLINE, PubMed, the Cochrane library, Embase, and the Web of Science. Statistical analysis was performed with OpenMetaAnalyst, MetaDiSc version 1.4, and Medcalc version 17.9.7.

**Results**: Five studies including 2480 patients were included. The sensitivity and specificity for narrow-band imaging were 0.90 (0.73-0.97) and 0.95 (0.81-0.99) respectively. The positive likelihood ratio and negative likelihood ratio were 18.82 (0.31-82.1) and 0.08 (0.02-0.31). For white light endoscopy, the sensitivity and specificity were 0.77 (0.58-0.89) and 0.91 (0.79-0.96). The positive likelihood ratio was 7.61 (3.61-16.04), and the negative likelihood ratio was 0.21 (0.11-0.39). The odds ratio for detection rates between narrow-band imaging and white light endoscopy was 4.29 (0.56-33.03, p = 0.16). Area under the curve for narrow-band imaging was 0.98 (SE: 0.02), and for white light it was 0.93 (SE: 0.03). There was no significant difference in the receiver operating characteristic curves between the two modalities (p = 0.14).

**Conclusion**: Narrow-band imaging showed a higher sensitivity and positive likelihood ratio for the diagnosis of nasopharyngeal carcinoma. However, there was no significant difference in detection rates compared to white light endoscopy. Further investigation with a uniform diagnostic criteria and terminology is needed for narrow-band imaging in the diagnosis of nasopharyngeal carcinoma.

## Introduction

Nasopharyngeal carcinoma (NPC) is a common head and neck cancer in the southeast Asia ^1^. The age-standardized incidence rate in Hong Kong is 12.6 per 100,000 for males and 3.9 per 100,000 for females ^2^. The current standard for NPC diagnosis is histological from a white light endoscopy (WL) directed biopsy ^3^. Large tumors are easy to identify. Early and small tumors might be impossible to differentiate from adenoidal tissue or normal nasopharyngeal mucosa ^4^.

Narrow-band imaging (NBI) is an imaging technique that uses two specific wavelengths of light that are strongly absorbed by hemoglobin, allowing improved visualization and delineation of mucosal microvascular patterns ^5^. This technique, which has been used for the detection of adenomas in the gastrointestinal tract, has the potential to reduce the false negative rates associated with conventional white light endoscopy ^6^. If the sensitivity of abnormal vasculature with the assumed overlying mucosal malignancy seen on NBI was able to surpass that of abnormal morphology of the nasopharynx seen on WL, the false negative findings would be reduced and unnecessary biopsies and their potential complications avoided ^7^.

NBI has been described in the early detection of other head and neck cancers, including squamous cell carcinomas (SCC) of the larynx, floor of mouth ^8^, oropharynx, and hypopharynx ^9^. Among these studies, the finding of brownish spots was the most common descriptive morphology followed by irregular vascular patterns. Similar NBI abnormalities have been adapted to identify primary NPC. The aim of this study was to use a meta-analysis to evaluate the diagnostic utility of NBI compared to conventional WL for the detection and diagnosis of NPC.

## Methods

### Eligibility and data extraction

**Figure 1.**
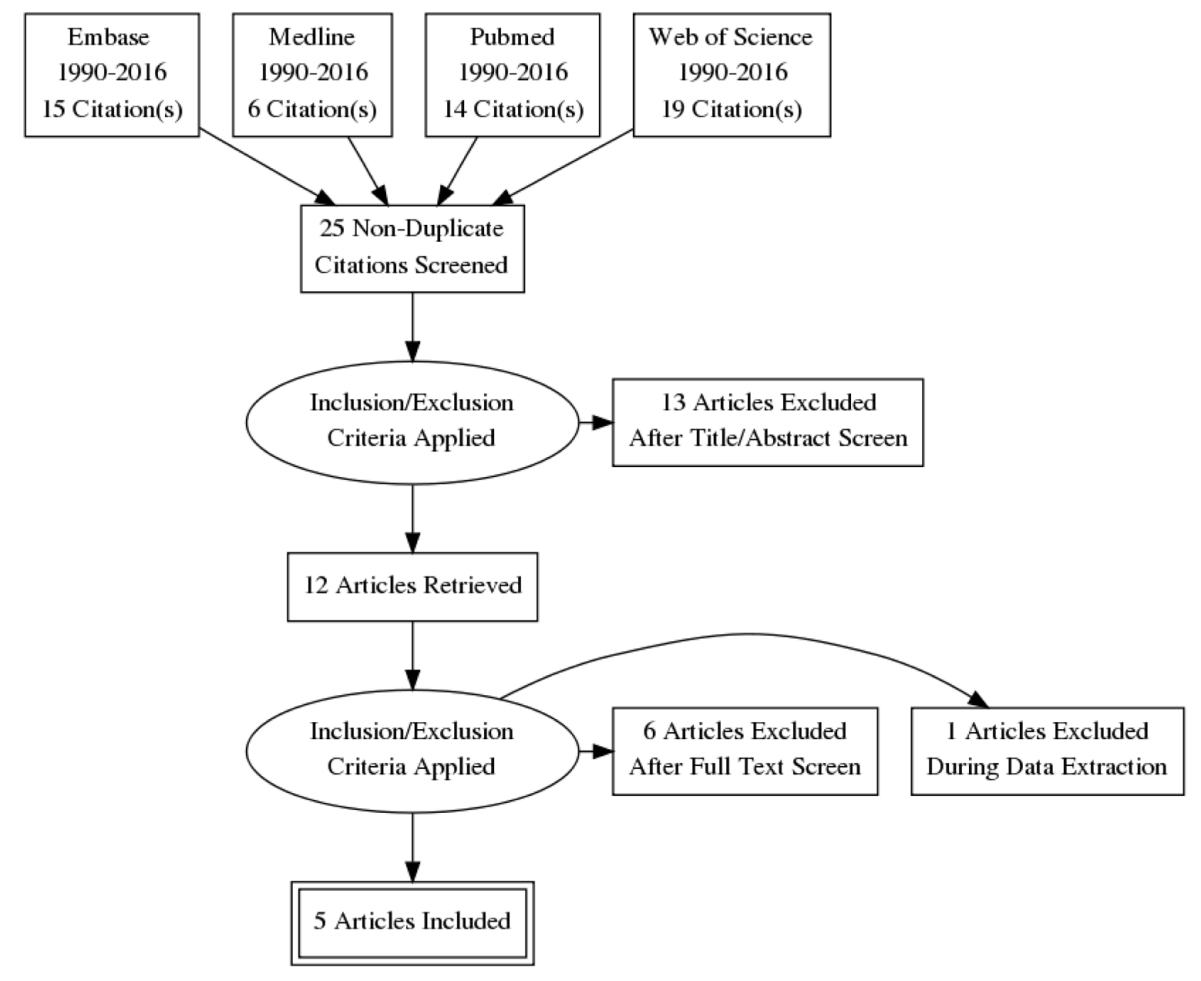
This PRISMA Chart was constructed to illustrate the workflow of the review process.

We included all prospective studies detecting NPC by using NBI compared with standard WL. Excluded studies were reviews, data reported only as abstracts, non-diagnostic studies, those that did not include histological confirmation or extractable raw data, and retrospective studies. The publications, their relevance, and eligibility were determined independently by DCMY and JYKC. Application of the inclusion and exclusion criteria was undertaken independently by both reviewers, and any difference of opinion was resolved by discussion between the reviewers. Data extraction was done by DCMY and JYKC. Included studies were assessed for quality. The PRISMA diagram is shown in Figure 1. The study was exempt from Institutional Review Board approval as no patient identifiable data was utilized.

### Search strategy

MEDLINE, PubMed, the Cochrane library, Embase, and the Web of Science were searched to identify studies in which narrow band imaging endoscopy was used to look for nasopharyngeal carcinoma compared with white light endoscopy. We used the search terms ‘narrow band imaging,’ ‘narrow band imaging vs white light imaging,’ and ‘nasopharyngeal carcinoma’. As an example, for MEDLINE, we searched the terms “Narrow Band Imaging” and “Nasopharyngeal Neoplasms” separately. We subsequently combined them as an “AND” search, yielding six articles for that specific database. We only included prospective trials of NBI versus standard WL. Only articles in English were included. Reviewers were not blinded to the names of authors, institutions, or journals. The reference lists of these articles were searched for additional relevant articles.

### Statistical analysis

A DerSimonian-Laird diagnostic random effects model was adopted for statistical analysis of sensitivity, specificity, positive and negative likelihood ratios for NBI and WL respectively. Detection rates, defined by true positives divided by sample size, were analyzed and compared between NBI and WL using a binary random effects model. Receiver operating characteristic (ROC) curves were constructed and compared with the Hanley and McNeil approach. Funnel plots were not constructed as the relatively small number of primary studies available for this meta-analysis would make it difficult to interpret ^10^. Statistical analysis was performed with OpenMetaAnalyst version 12.11.14; ROC curves and meta-regression were performed using MetaDiSc version 1.4; ROC curve comparison analysis was performed with Medcalc version 17.9.7.

## Results

A total of 2480 patients, 61% male and 39% female, were included in our meta-analysis. The mean patient age was 49.5 years. No range was calculated for age and sex as not all studies had included them. Basic demographics are listed in Table 1. The indications for nasoendoscopy in the studies are shown in Table 2. Details of endoscopic examination specifics of the included studies are listed in Table 2. A total of 191 patients were diagnosed with NPC. NBI and WL successfully detected 191 and 163 of these cases respectively.

**Table 1.** Demographics of included studies.

Study	Country	Patients	Male (%)	Female (%)	Mean age
Vlantis 2016	Hong Kong	156	90 (58%)	66 (42%)	49.5
Wang 2011	Taiwan	79	58 (73%)	21 (27%)	52.9
Wang 2012	Taiwan	106	80 (75%)	26 (25%)	55.6
Wen 2012	Guangzhou, China	285	133 (47%)	152 (53%)	38
Yang 2012	Guangzhou, China	1854	1153 (62%)	701 (38%)	53.1

**Table 2.** Endoscope examination characteristics.

Study	Endoscopic examination purpose	Endoscope (Olympus medical system)	Light source (Olympus medical system)	Video system (Olympus medical system)	NBI abnormality
Vlantis 2016	Screening	ENF-VQ	CLV-S40 PRO	Visera OTV-S7 PRO	Vascular tufts, dilated, and enlarged vessels
Wang 2011	Screening or surveillance for recurrence	ENF-V2 or VQ	CLV- 160B	CV-160B	Irregular microvascular pattern and side differences including Light crests
Wang 2012	Surveillance for recurrence	ENF-V2 or VQ	CLV- 160B	CV-160B	Well demarcated brown spots and scattered brown spots
Wen 2012	Screening	A500	CLV-S40	CV-160B	Well demarcated brown spots, vessel Irregularity
Yang 2012	Screening	ENF-VT2	CLV-S40Pro	CV 160B	Well demarcated brownish area and scattered brown spots, irregularity of vessels

NBI – Narrow-band imaging

**Figure 2.**
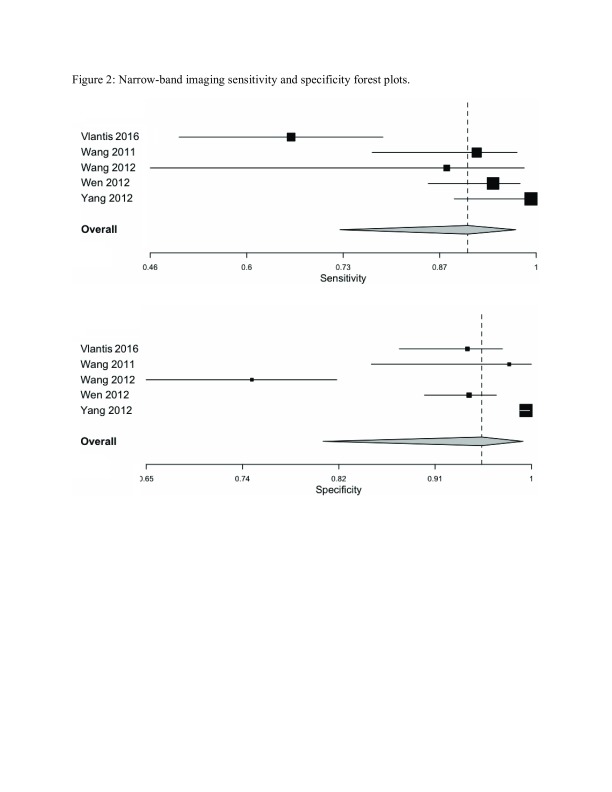
Narrow-band imaging sensitivity and specificity forest plots summarized the individual as well as the pooled sensitivities and specificities for nasendoscopy with narrow band imaging settings.

The pooled sensitivity and specificity for NBI was 0.90 (0.73–0.97) and 0.95 (0.81–0.99) respectively as shown in Figure 2. The ROC curve is shown in Figure 4 and has a calculated area under the curve (AUC) of 0.98 (SE: 0.02). The pooled positive likelihood ratio and negative likelihood ratio was 18.82 (4.31–82.06) and 0.08 (0.02–0.31). The pooled diagnostic odds ratio for NBI was 200.13 (32.56–1230.33, p < 0.001) with tau^2 3.34, Q(df=4) 23.90, hetergeneity p-value < 0.001, and I^2 being 83.26.

**Figure 3.**
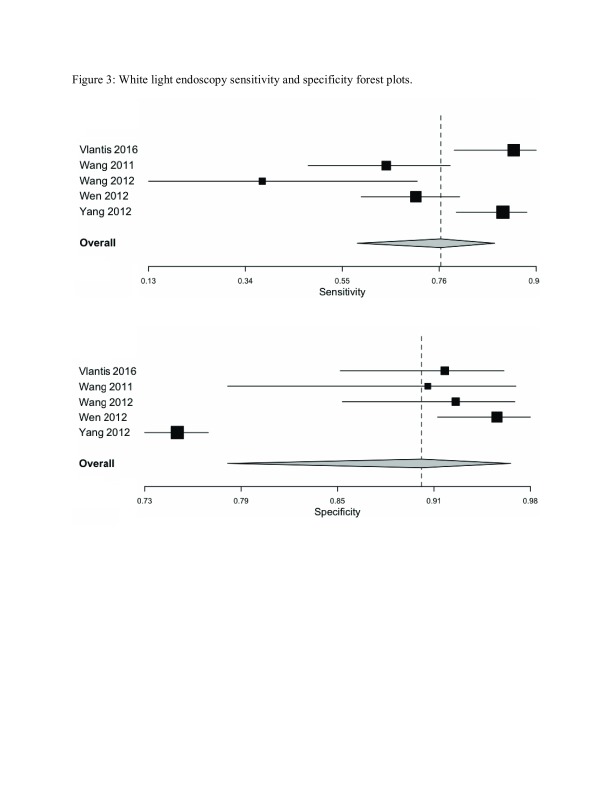
White light endoscopy sensitivity and specificity forest plots summarized the individual as well as the pooled sensitivities and specificities for nasoendoscopy with conventional white light settings.

**Figure 4.**
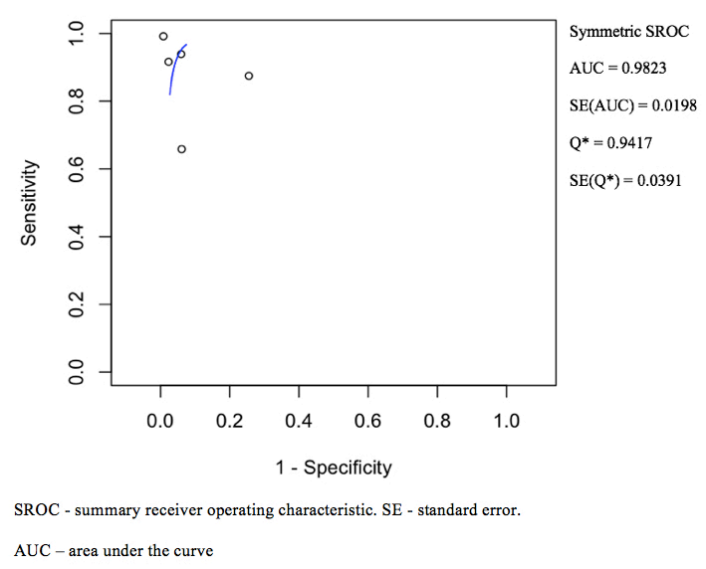
Narrow-band imaging summary receiver operating characteristic curve illustrated the accuracy of nasoendoscopy with narrow-band imaging settings. Area under the curve and standard error was calculated.

**Figure 5.**
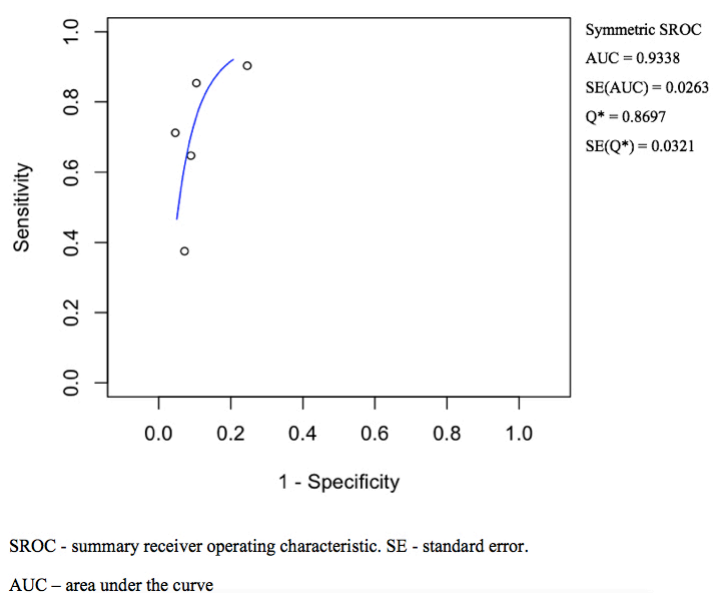
White light summary receiver operating characteristic curve illustrates the accuracy of nasoendoscopy with conventional white light settings. Area under the curve and standard error was calculated.

For WL, the pooled sensitivity and specificity was 0.77 (0.58–0.89) and 0.91 (0.79–0.96) as shown in Figure 3 respectively. The ROC curve is shown in Figure 5, and the AUC calculated as 0.93 (SE: 0.03) The pooled positive likelihood ratio is 7.61 (3.61–16.04) and the negative likelihood ratio is 0.21 (0.11–0.39). The pooled diagnostic odds ratio is 34.00 (15.58–74.21, p < 0.001) for WL, with tau^2 0.45, Q(df=4) 9.67, hetergeneity p-value: 0.046, I^2 being 58.63. A summary of pooled statistics and analyses is depicted in Table 3.

**Table 3.** Narrow-band imaging and white light endoscopy pooled data and analysis.

	NBI	WL
Sensitivity	0.90 (0.73–0.97)	0.77 (0.58–0.89)
Specificity	0.95 (0.81–0.99)	0.91 (0.79–0.96)
Positive Likelihood Ratio	18.82 (4.31–82.06)	7.61 (3.61–16.04)
Negative Likelihood Ratio	0.076 (0.018–0.31)	0.21 (0.11–0.39)
Diagnostic Odds Ratio	200.13 (32.56–1230.33)	34.00 (15.58–74.21)
AUC	0.98 (SE: 0.02)	0.93 (SE: 0.03)

NBI - Narrow-band imaging. WL - white light endoscopy. AUC – area under the curve. SE – standard error.

For heterogeneity analysis, meta-regression was performed to identify the source of heterogeneity for the following factors: number of patients, percentage of males or females and mean age. However, none of them accounted for heterogeneity in either group.

In the analysis of detection rates between NBI and WL, the odds ratio was 4.29 (0.56–33.03, p = 0.16), the Tau^2 was 4.35, Q(df=4) was 25.39, heterogeneity p value was <0.001, and I^2 was 84.24. There was no significant difference between detection rates of NBI and WL. Comparing the ROC curve between and NBI and WL, there was no significant difference (p = 0.14).

Dataset 1. OpenMetaAnalyst file contain data analysis performed in this study http://dx.doi.org/10.5256/f1000research.15183.d206977
Copyright: © 2018 Yeung DC et al. 2018 Data associated with the article are available under the terms of the Creative Commons Zero "No rights reserved" data waiver (CC0 1.0 Public domain dedication).

## Discussion

In this meta-analysis comparing NBI to WL for the detection and diagnosis of primary nasopharyngeal carcinoma, our study found that NBI had a higher specificity, sensitivity, and positive likelihood ratio. However contrary to previous studies, there were no significant differences between NBI and WL for sensitivity analyses and detection rates. Both tests had similar accuracies as indicated by an AUC approaching the value of 1. This likely reflects the fact that WL is an established examination to evaluate the nasopharynx, and that there are no significant advantages of using current otolaryngological NBI systems to detect NPC, perhaps also indicative of the lack of magnification that is available with larger diameter gastrointestinal endoscopes but not with the smaller nasopharyngeal endoscopes.

Early detection of NPC is important given the differences in treatment regimens and prognoses for early versus late NPC. Modalities useful in the screening, diagnosis and staging of primary NPC that supplement nasoendoscopy including MRI, CT, PET-CT, and plasma Epstein-Barr virus (EBV) DNA ^11^. However, one or more of these may not always be readily available, may be time consuming, and may be costly in the routine diagnosis of NPC. Plasma EBV DNA has recently been shown to be a highly sensitive and specific screening tool for NPC ^1^, but again the technology to assess plasma EBV DNA has not been standardized to make this a definitively useful investigation. For these reasons, NBI has the potential to be useful by improving the endoscopic detection of primary NPC.

Endoscopes used in the examination of the nasopharynx are usually 4mm in diameter, unlike gastrointestinal endoscopes which are 9 to 12mm in diameter. As current NBI endoscopes are distal sensing endoscopes, the smaller diameter limits the size of the distal sensing chip at the tip of the endoscope, thus limiting the pixel density and resolution and thus the ability to detect smaller lesions ^12^. The endoscopes used in this study might not have had sufficient magnification to observe the microvascular patterns of the nasopharynx in sufficient detail when compared to gastrointestinal endoscopy ^13^. With the advance of ultra-high definition distal chips now offering a resolution of up to 4k, and with 8k resolution under development ^14^, the utility of NBI in the detection and diagnosis of primary NPC may improve significantly.

A further potential issue with NBI being used as a screening tool for the detection of NPC is that NBI endoscopy requires specific training and there is a learning curve. NBI images are initially exceptionally difficult to interpret, and without uniform diagnostic criteria, are not particularly helpful. The interpretation of abnormal features such as vascular tufts or tortuous vessels could theoretically affect accuracy. One concern is that NBI might lead to an increased number of unjustified biopsies due to false positive findings of NBI abnormalities ^15^. NBI was however shown to have a high specificity of 0.95 in our study. This could be either due to the fact that the endoscopists included in this study were already well trained and experienced, or that the learning curve was less of a problem than was postulated.

Most of the papers included in this study primarily focused on what they termed brownish spots as the predominant NBI detected abnormality, which was felt to represent a macroscopic focal increase in subepithelial microvascular architectural density ^16– 18^. Terms of vascular patterns such as vessel tortuosity, dilation, and irregularity followed. The utilization of other mucosal surface structural abnormalities in the epithelial layer was only mentioned in one study which included light crests and side morphological differences detected by NBI ^19^. In our colorectal counterparts, a universal NBI magnifying endoscopic classification of colorectal tumors based on objective grounds using a modified Delphi method, followed a proposal by the Japanese NBI Expert Team. They classified abnormal NBI findings into four categories based on the vascular pattern. Mucosal surface patterns were included in this classification: dark or white spots; tubular, branched, and papillary; irregular or obscure; and amorphous areas ^20^. Mucosal surface patterns of oval, tubular, papillary, and destructive were described in histological confirmed gastric carcinomas ^21^. One example of the utilization of mucosa surface structural abnormalities in the head and neck region was in a study of NBI on laryngeal squamous cell carcinoma ^9^. The sensitivity and specificity of NBI was described to be both 0.91 respectively. Mucosal abnormalities detected with NBI were demarcated brownish areas with scattered brown spots in the lesion on the epiglottis. In the nasopharynx, the most common type of epithelial malignancy is a non-keratinizing undifferentiated carcinoma. Although the sensitivity and specificity was 0.90 and 0.95 in our meta-analysis, adopting a uniform epithelial abnormality classification similar to colorectal and upper gastrointestinal diagnostics might be a suitable step in optimizing NBI for the detection of NPC.

Furthermore, solely using vascular patterns to differentiate malignant from benign lesions may be difficult in practice. In a paper investigating the difference between benign basal cell hyperplasia (BCH) and head and neck SCC, BCH was described as having a regular distribution of capillary loops and preserved intervascular transparency compared to SCC. However, no significant differences were detected in the sharpness of the lesion border, nor in the dilatation and tortuosity of the capillary loops ^22^. If every lesion showing dilatation and tortuosity of capillary loops were to be biopsied, it would defeat one aim of NBI and that is to decrease the number of unjustified biopsies.

Limitations of the current analysis include the heterogeneity between primary studies that limit the accuracy of this meta-analysis. These variations include inclusion and exclusion criteria, indications for nasoendoscopy, operator experience, interpretation of endoscopic findings, and diagnostic thresholds. Convenience samples of available examiners and power calculations were not included in any of the studies to calculate the number of examiners needed to detect significant differences. Only studies written in English were included in this meta-analysis. Other languages may offer primary studies with larger sample sizes. Finally, the inclusion of examiners with high baseline detection rates but with little potential to improve may also have limited the effect sizes.

## Conclusion

For the detection of primary nasopharyngeal carcinoma, narrow-band imaging has not been shown to have significant advantage over white light endoscopy in this meta-analysis, which may be related to the heterogeneity of studies analyzed. Detection may be improved with uniform diagnostic criteria and the inclusion of additional definitions and patterns of mucosal microstructures and submucosal microvascular abnormalities.

This work was previously presented at the IFOS World Congress of Otorhinolaryngology and Head and Neck Surgery on 28 June 2017, Paris, France.

## Data availability

The data referenced by this article are under copyright with the following copyright statement: Copyright: © 2018 Yeung DC et al.

Data associated with the article are available under the terms of the Creative Commons Zero "No rights reserved" data waiver (CC0 1.0 Public domain dedication). 

Dataset 1: OpenMetaAnalyst file contain data analysis performed in this study. 10.5256/f1000research.15183.d206977 ^23^

## Supplementary material

Supplementary File 1 – Completed PRISMA checklist.
