# Supplementary material for: A meta-analysis of narrow-band imaging for the diagnosis of primary nasopharyngeal carcinoma
Source: F1000Res. 2018 Jun 18;7:759. [Version 1] doi: 10.12688/f1000research.15183.1 (PMC6039959; doi:10.12688/f1000research.15183.1)
Supplement: Peer review history

# Peer review: Referee response for version 1

**DOI:** 10.5256/f1000research.16541.r35278

**Published:** 2018-07-10

**Authors:** Daniel Clayburgh, Jennifer Christenson

**Affiliations:** 1 Department of Otolaryngology/Head and Neck Surgery, Oregon Health and Science University, Portland, OR, USA

In this manuscript, Yeung *et al *perform a meta-analysis of narrow band imaging versus white light endoscopy for the diagnosis nasopharyngeal carcinoma.A few areas that could be improved in this report:

 Introduction

 Explain more about how narrow band imaging works and detects mucosal change.

 Methods

 Included studies inclusion criteria are very loosely explained (discussed between DCMY and JYKC), No specific criteria other than prospective studies that compare NBI to WL.  Please provide more detail on study protocols that were used.

 Provide additional detail on imaging characteristics that were concerning for neoplastic change and warranted biopsy.

 Results:

 Table 1 and 2 could easily be combined in to a "study characteristics" table. 

 Discussion

 One confounding factor would be in the indications for imaging and/or biopsy.  Mucosal appearance of screening patients and surveillance patients are likely very different aka post-treatment/ radiated mucosa.

We have read this submission. We believe that we have an appropriate level of expertise to confirm that it is of an acceptable scientific standard.

# Peer review: Referee response for version 1

**DOI:** 10.5256/f1000research.16541.r35284

**Published:** 2018-07-09

**Authors:** Chwee Ming Lim

**Affiliations:** 1 Department of Otolaryngology, Head and Neck Surgery,, National University of Singapore, Singapore, Singapore

A comprehensive review/meta-analysis on the topic of NBI in diagnosis of NPC. Several comments that i have are

 1) PRISMA chart - Figure 1

 The authors should expand on the reasons of the exclusion of the papers. Having 13 papers excluded after title screen, one paper excluded after data collection without clarification may leave the readers wondering on the nature of the exclusion.

 2) The heterogeneity analysis is an  inherent weakness of this study which the authors have addressed in the discussion. Having NBI in separate clinical context ( ie screening and if so, in specific high risk cohorts versus general population; and surveillance cohort should be explored). Can a separate robust analysis be performed on the use of NBI in these specific clinical scenarios with the existing dataset?

 3) A recent meta-analysis published in Otolaryngology-Head and Neck Surgery on this subject yielded similar conclusions. The authors may want to highlight some similarities or differences in their analysis and approach.

 Overall, an informative article on the subject matter and reviewing the existing evidence of NBI in diagnosis of NPC.

I have read this submission. I believe that I have an appropriate level of expertise to confirm that it is of an acceptable scientific standard.
